# Xanthogranulomatous Pyelonephritis Presenting with a Left Flank Mass

**DOI:** 10.1155/2013/362194

**Published:** 2013-12-03

**Authors:** Uwais B. Zaid, Sima P. Porten, Nadya M. Cinman, Thomas H. Sanford, Benjamin N. Breyer

**Affiliations:** Department of Urology, University of California San Francisco, 400 Parnassus Avenue, San Francisco, CA 94143, USA

## Abstract

We present a case of a patient with xanthogranulomatous pyelonephritis (XGP) presenting with a large (18 × 12 cm) left-sided flank mass with worsening left flank pain. CT abdomen/pelvis demonstrated a left kidney with parenchyma replaced by multiple large hypodense collections containing fluid and gas, a left staghorn calculus, and a communication between the kidney and large flank collection. About 4.5 weeks after initial presentation, the patient underwent operative intervention. Pathology revealed an end-stage kidney with scar consistent with xanthogranulomatous pyelonephritis.

## 1. Case Presentation 

**Figure 1:**
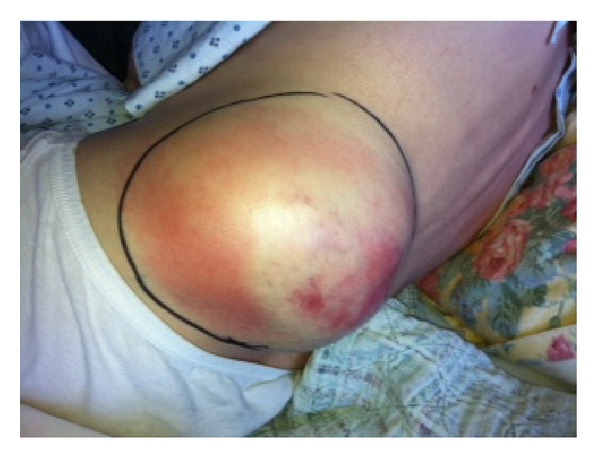
Large left 18 × 12 cm tender fluctuant flank mass with blanching erythema.

A 59-year-old woman presented to our institution with one week of progressively worsening left flank pain and massive left flank mass readily apparent on exam. She denied fevers, chills, hematuria, dysuria, or weight loss. She did endorse 1 week of nonbilious, nonbloody emesis. Her pain was sharp with anterior radiation. She denied tobacco and illicit drug use, recent travels, and sick contacts. She denied previous surgeries or medical problems. At initial presentation, her vitals were within normal limits. Notably, she did not have a fever. On examination, we noted a large 18 × 12 cm soft and tender left flank mass with blanching erythema and fluctuance (Figure 1).

We obtained serum laboratories, which revealed a white cell count of 15 × 10^9^/L, hematocrit of 31.1%, creatinine of 1.26 mg/dL, alkaline phosphatase of 180 U/L, erythrocyte sedimentation rate of 146 mm/hr, and INR of 1.5 s. A urine analysis demonstrated positive nitrites, 5–10 WBC/hpf, 2–5 RBC/hpf, and negative leukocyte esterase. Urine culture grew out 100,000 CFU/mL of *Escherichia coli*. The remainder of her liver function panel, basic metabolic panel, and CEA were within normal limits.

**Figure 2:**
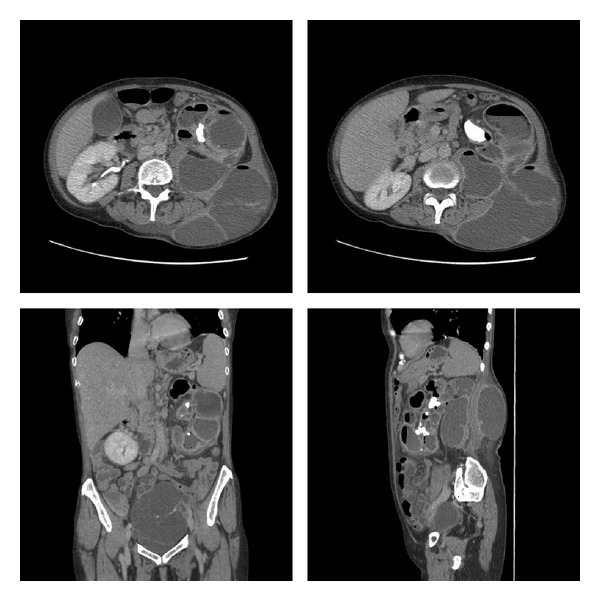
CT abdomen/pelvis with IV contrast and delayed phase demonstrating a left kidney with parenchyma replaced with multiple large hypodense collections containing fluid and gas, a left staghorn calculus, and a communication between the kidney and large flank collection. Additionally, a 13.5 × 7.7 cm multiloculated pelvic mass was noted.

A CT abdomen/pelvis with IV contrast and delayed phase (Figure 2) demonstrated a left kidney with parenchyma replaced by multiple large hypodense collections containing fluid and gas, a left staghorn calculus, and a communication between the kidney and large flank collection. Additionally, a 13.5 × 7.7 cm multiloculated pelvic mass was seen. The classic “bear paw print” appearance characterized by low attenuating cystic areas with a central stone [1] was not clearly visualized. Our colleagues in interventional radiology placed ultrasound guided percutaneous drainage tubes, which drained 600 cc of purulent fluid. The output was sent for creatinine, which was normal, cytology, which was benign, and cultures, which grew out *E. coli*, *Proteus mirabilis*, and *Streptococci viridans* but were negative for acid-fast bacilli.

**Figure 3:**
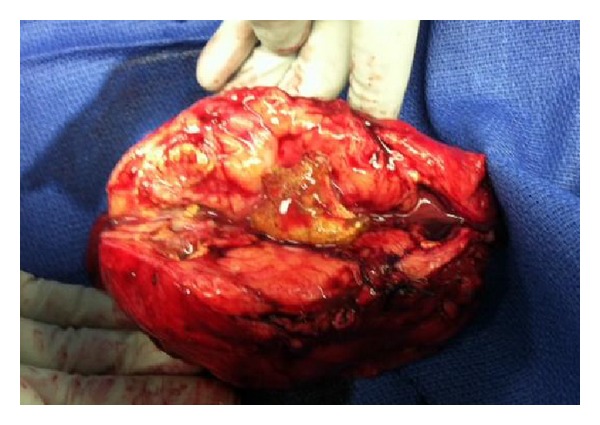
Intraoperative photograph of left XGP kidney.

About 4.5 weeks after initial presentation, the patient underwent left open adrenal sparing nephrectomy, splenorrhaphy, repair of transverse colonic fistula, and left salpingo-oophorectomy (Figure 3). Pathology revealed an end-stage kidney with scar and foamy lipid laden macrophages consistent with xanthogranulomatous pyelonephritis. The incidentally noted left ovarian tumor was a multiloculated mucinous cystadenoma with numerous macrocalcifications.

## 2. Discussion

Xanthogranulomatous pyelonephritis (XGP) is a severe and chronic renal infection associated with diffuse renal destruction most commonly from obstructive uropathy. This is an uncommon type of infective pyelonephritis marked with destructive changes to renal parenchyma and surrounding tissues, most commonly resulting from obstructive uropathy associated with nephrolithiasis. Our patient did have an incidentally noted pelvic mass, which may have led to obstruction of her left renal unit with subsequent development of nephrolithiasis. XGP is more commonly seen in middle aged women. Additional risk factors include diabetes, recurrent urinary tract infections (UTIs), and hyperlipidemia. Typically, patients present with fevers, malaise, weight loss, and urine cultures growing out *Escherichia coli*, *Proteus mirabilis*, and *Pseudomonas aeruginosa* may be polymicrobial. Laboratory abnormalities are often nonspecific, with anemia, leukocytosis, elevated erythrocyte sedimentation rate (ESR), and abnormal liver function tests [2–5]. Such was the case in our patient who had a left-sided staghorn calculus, a polymicrobial culture, elevated WBC, low hematocrit, elevated ESR, and elevated alkaline phosphatase.

Diagnosis is often by CT scan, which may also differentiate XGP from emphysematous pyelonephritis [5]. The classic finding on CT with IV contrast is the “bear paw sign,” marked by a centrally obstructing stone with low-attenuating surrounding hydronephrotic parenchyma [1]. Additionally, series have shown that poor functioning or functionless kidneys were seen in 83% of patients presenting with XGP, implying delayed presentation [5]. Management may be via open or laparoscopic nephrectomy, however, with the later reported rates of open conversion range from 16 to 33% and complication rate of 20–50% [3, 6]. Pathology shows lipid-laden macrophages with granulomatous infiltration [4, 5, 7].
